# The impact of healthcare reform on the dynamic changes in health service utilization and equity: a 10-year follow-up study

**DOI:** 10.1038/s41598-022-07405-y

**Published:** 2022-03-04

**Authors:** Wenqin Guo, Gangjun Liu, Li Ma, Baokai Gao, Wenlong Wang, Zhaoyan Hu, Yanmei Tian, Wenwen Xiao, Hui Qiao

**Affiliations:** 1 School of Public Health and Management, Ningxia Medical University, Yinchuan, China; 2 Key Laboratory of Environmental Factors and Chronic Disease Control, No.1160, Shengli Street, Xingqing District, Yinchuan, Ningxia China

**Keywords:** Health care, Medical research

## Abstract

In the past decade, the government of China has implemented healthcare reforms to provide universal access to healthcare by 2020. We aimed to systematically analyse the dynamic changes in health services and equity during the past 10 years to understand the correlation between health services and social-economic status. We performed a longitudinal study in which we extracted aggregated data mainly from a project (2009, 2011, 2012, 2015, 2019). A multi-stage stratified cluster randomized design was used to obtain a representative sample in each county. Concentration indexes were used to analyse the equity of the changes in utilization. We built multivariate random-effects generalized least squares regression models with the panel data to test whether the rate of receiving a medical consultation in the last 2 weeks or the rate of hospital admission or the prevalence of chronic illness was associated with social-economic status including education level and rural disposable income per capita. We found declines in both the rate of not receiving a medical consultation during the last 2 weeks (P < 0.05 intervention group) and the rate of hospital avoidance (P < 0.05) from 2009 to 2019. The equity in residents' health service utilization has improved constantly. We additionally found that rural disposable income per capita is a protective factor for the rate of a receiving a medical consultation during the last 2 weeks and the rate of hospital admission. China’s 2009 healthcare reform have positively influenced utilization rates and equity in health service utilization in the past decade, a range of health service-targeted strategies are needed including strengthen the prevention and treatment of chronic diseases, focus attention on the health status of elderly residents and improve social-economic status, especially the level of education.

## Introduction

Health equity is a basic value assumed in the health policies of many countries worldwide^1^. In April 2009, the Chinese government issued a new healthcare reform plan^2^ meant to provide equity, safe, effective, medical services for residents. An additional 850 billion yuan (approximately US $125 billion) was planned for 2009–2011, with the aim of providing basic healthcare that is accessible and equitable to residents by 2020^3^. China's total health expenditure increased 3.8 times from 2009 to 2019^4^, and the number of health technicians per thousand people (number of licensed doctors or licensed assistant doctors and registered nurses) increased from 4.15 to 7.26. China has made great progress in achieving equity in health services^5^. Song found that the largest and fastest growth of the number of health professionals was in western-non-poor counties^6^. Fu found that a systemic reform, where payment system is aligned, can significantly improve the performance of public hospitals^7^. Chai et al. found that scale efficiency has improved after healthcare reform, but low educational attainment and higher percentage of out-of-pocket payments had adverse effects^8^. The healthcare reform reduced the disease burden of residents^9,10^. The coverage of the new rural cooperative medical system has been expanded to almost all rural residents, which makes the utilization of inpatient health services more equitable^11^. Winnie et al. found that from 2010 to 2016, the hospital admission rate increased 1.8 times and the probability of seeing a doctor in the last 2 weeks increased 1.4 times^12^.

In 2009, a project named ‘Innovating payment system and improving health benefits’, which was jointly carried out by Harvard University, Oxford University, Fudan University and Ningxia Medical University, was implemented in Ningxia, China^13,14^. This project mainly focused on adjusting the healthcare payment system. Haiyuan County and Yanchi County in the Ningxia Hui Autonomous Region were selected as the sample counties in which the project was carried out^15^. The intervention in the reform of county payment system is mainly aimed at the root cause of the imbalance of incentive and restraint mechanisms between the supply and demand of medical services. Technically, it aims to improve the outpatient reimbursement proportion of NCMS to grass-roots medical institutions, and implement the performance-based capitation prepayment for the outpatient services provided by township health centers and village clinics as a whole, so as to properly balance the increase of village medical income. The economic incentive based on the improvement of the accessibility and ability of grass-roots services, and the reduction of the burden of medical expenses have formed a mechanism innovation scheme with the reform of payment system as the core content. How effective is the implementation of this health care reform project?

In this study, we use data from the survey of rural residents from 2009, 2011, 2012, 2015 and 2019 to explore the influence of healthcare reform on improvements in health service demand, utilization and equity, and the correlation between socio-economic factors and health service utilization. We hypothesize that the increase in health service utilization over the past 10 years occurred in groups of different socio-economic status and that important advances have been made in achieving equal access to services.

## Methods

### Study design and sample

Data were obtained mainly from the project ‘Innovating payment system and improving health benefits’, which was jointly carried out by Harvard University, Oxford University, Fudan University and Ningxia Medical University (2009, 2011 and 2012). The data from 2015 and 2019 were extracted from the National Natural Science Foundation of China (from a follow-up study of project). Questionnaires were completed and verified by trained teachers and graduate students. The survey was answered by one adult per household who provided information on all members.

A multi-stage stratified cluster randomized design was used to obtain a representative sample from each county. We selected a total of five counties in Ningxia Province, including two project counties (Haiyuan and Yanchi) and three control counties (Tongxin, Pengyang and Xiji). In each county, all the villages were divided into three economic levels, 40% of the sample villages were selected. Then, using the household head roster, 33 households (20 households in the control counties) in each village were selected by systematic sampling. Members of the sample households who had been living there for more than 6 months were selected as respondents. The survey conducted in the baseline year (2009) included 30,384 observations, and there were 28,886, 30,583, 28,697, and 23,821 observations in follow-up years 2011, 2012, 2015, and 2019, respectively . Sample data for Tongxin county is missing in 2019.

### Concentration index

Equity in inpatient and outpatient utilization: The concentration index (CI) was used to measure equity in healthcare use. The CI is one of the most commonly used indexes to measure health service equity.

The specific steps of calculating the concentration index are as follows: ① sorting by economic income group and giving the relevant rank X (0–1); ② calculating the health level or disease prevalence rate h of each economic income group according to economic income; ③ calculating the average level M of health or disease of the whole population (such as 2-week prevalence rate and hospitalization rate in the past year); ④ calculating the correlation rank X and each economic income group (5) calculation of concentration index^16^:

$${\text{CI }} = {\text{ 2cov }}\left( {{\text{X}},{\text{ H}}} \right)/{\text{M}}$$

$${\text{cov }}\left( {{\text{X}},{\text{ H}}} \right) \, = {\text{E}}\left( {{\text{XH}}} \right) \, - {\text{ E}}\left( {\text{X}} \right){\text{ E}}\left( {\text{H}} \right)$$

X is the relevant rank given by economic income grouping, H is the health level or disease prevalence calculated by different economic income grouping, and M is the average level of health or disease of the whole population (such as 2-week prevalence and hospitalization rate in the past year). CI was between − 1 and 1. For health CI, "CI = − 1" means that health is concentrated in the hands of the people with the lowest socio-economic status group, "CI =  + 1" means that health is concentrated in the hands of the people with the highest socio-economic status group; for disease CI, if it is negative, it means that disease is concentrated in the hands of the lower socio-economic status group, and disease CI is positive, it means that disease is concentrated in the hands of the higher socio-economic status group. When CI tends to 0, it indicates that the distribution of health status or diseases is fair.

### Statistical analysis

In this study, the sample data were checked for missing data and outliers and were cleaned prior to data analysis. We used scatter plots and Spearman’s rank correlation coefficient to assess the association between the disposable income of rural residents per capita or their educational level (represented as the proportion of people with an education level of senior high school or higher) and the chronic illness prevalence rate or hospital admission rate.

In the evaluation of health equity, socio-economic grouping is commonly used. Education level and income level are the main indicators to reflect the socio-economic situation^1^. We constructed multivariate random-effects generalized least squares regression models with our panel data to test whether the rate of receiving a medical consultation in the last 2 weeks or the rate of hospital admission was associated with education level, rural disposable income per capita, or the chronic illness prevalence rate. In those models, the group variable was county, and the time variable was year. The SE were adjusted for the clustering at the province level. The dependent variables were the rate of receiving a medical consultation in the last 2 weeks and the rate of hospital admission. The three independent variables were defined as follows: the percentage of individuals who had attended senior secondary school, which is the proportion of people with a senior secondary education level; per capita disposable income, which is the income that residents can freely spend; and the prevalence of chronic disease, which is the ratio of the number of individuals with a chronic illness surveyed in the first half of the year to the total number of people surveyed. The linear models included county data for 2009, 2011, 2012, 2015 and 2019. Tongxin County was excluded from the models for 2019 because of missing data. Our threshold for statistical significance was 0.05. We used Stata Version 14.0 for the analysis.

### Ethics approval and consent to participate

Ethical approval was granted (the Ethics Committee of Ningxia Medical University, Approval number, No. 2018-114). All methods were carried out in accordance with the relevant standards and regulations. All the residents (including all members of the family) of the sample households were invited to participate in the investigation, and the questionnaire was answered by the head of the household or another adult in the household. Informed consent was signed by the head of the household or an adult in the family. Each family only needed to sign one informed consent form.

## Results

### Potential health service needs were found, and the degree of unused health services decreased

**Figure 1:**
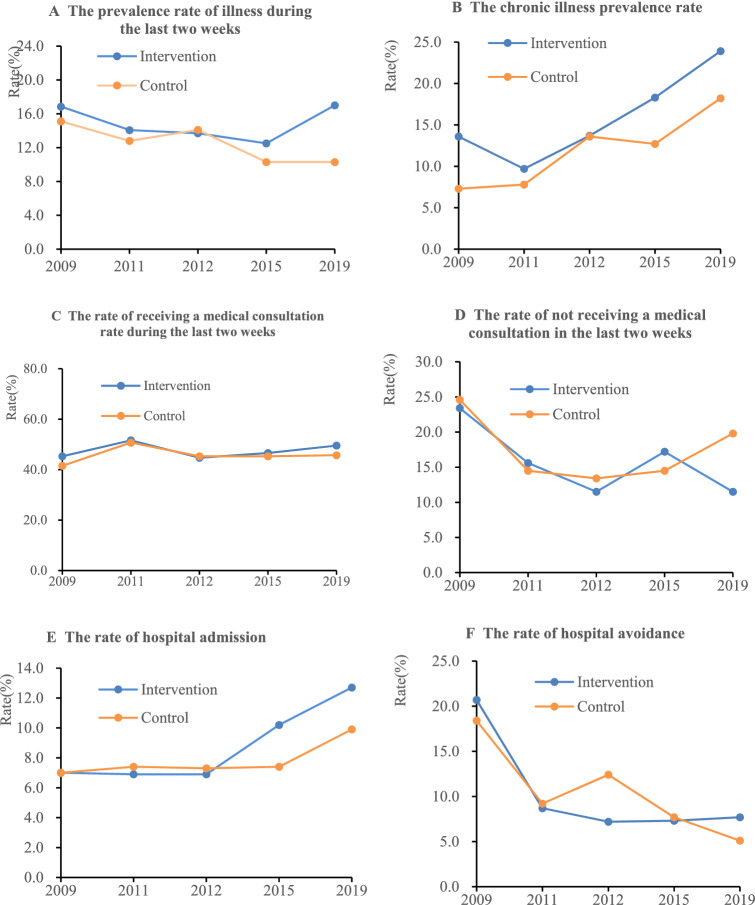
Changes in the need for and utilization of health services among rural residents in Ningxia, China, from 2009 to 2019.

Figure 1 shows the changes in the demand for and utilization of health services over the past 10 years in the rural areas of Ningxia, China. The prevalence rate of illness during the last 2 weeks increased from 16.9 to 17.0% (P > 0.05) in the intervention group, and the rate declined from 15.1 to 10.3% (P > 0.05) in the control group. The chronic illness prevalence rate increased from 13.6 to 23.9% (P > 0.05) in the intervention group, and it increased from 7.3 to 18.2% (P = 0.019 < 0.05) in the control group. The rate of receiving a medical consultation in the last 2 weeks increased from 45.3% to 49.5% (P > 0.05) in the intervention group and from 41.5 to 45.7% (P > 0.05) in the control group. The rate of not receiving a medical consultation during the last 2 weeks decreased from 23.4 to 11.5% (P = 0.038 < 0.05) in the intervention group and declined from 24.6 to 19.8% (P > 0.05) in the control group. The rate of hospital admission increased from 7.0 to 12.7% (P > 0.05) in the intervention group and increased from 7.0 to 9.9% (P > 0.05) in the control group. The rate of hospital avoidance decreased from 20.7 to 7.7% (P = 0.009 < 0.05) in the intervention group, and it declined from 18.4 to 5.1% (P = 0.004 < 0.05) in the control group.

**Figure 2:**
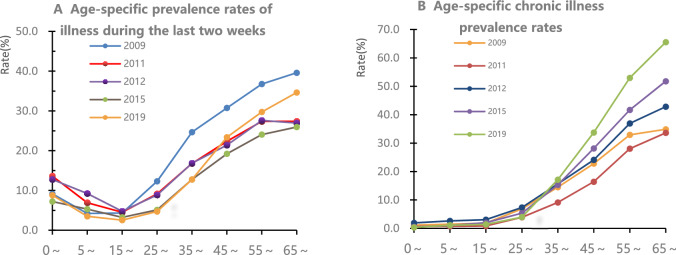
The age-specific prevalence rates of illness during the last 2 weeks and the age-specific chronic illness prevalence rates from 2009 to 2019.

Figure 2 shows the age-specific prevalence rates of illness during the last 2 weeks and the age-specific chronic illness prevalence rates from 2009 to 2019. Among 0- to 15-year-olds, the prevalence rate of illness during the last 2 weeks trended downward between 2009 and 2019. Among 15- to 65-year-olds, the prevalence rate of illness during the last 2 weeks showed an increasing trend with age, and the rate was highest in 2009. The age-specific chronic illness prevalence rates showed an increasing trend with age and time between 2009 and 2019. The total chronic illness prevalence rate increased from 10.8% in 2009 to 21.8% in 2019.

### More equitable use of health services among different socio-economic groups

Table 1 show the dynamic changes in the equity of health service utilization among rural residents in different income groups from 2009 to 2019. The absolute value of the concentration index for the rate of visiting a medical professional in the last 2 weeks decreased from 0.0312 in 2009 to 0.0105 in 2019, indicating that the equity in rural residents' outpatient health services utilization has improved. The absolute value of the concentration index for the hospitalization rate decreased from 0.0146 in 2009 to 0.0073 in 2019, and the absolute value of the concentration index tended towards 0, indicating that the utilization of inpatient health services among rural residents became increasingly equitable.

**Table 1:** Dynamic changes in the equity of health service utilization in different income groups from 2009 to 2019.

Year	Income quintile	Number of respondents	Number of patients	Number of visits	Number of inpatients	Visit rate	Hospitalization rate
2009	I	6273	1111	447	464	40.2	7.4
	II	5441	849	345	381	40.6	7.0
	III	6506	998	460	436	46.1	6.7
	IV	5983	996	446	413	44.8	6.9
	V	6181	929	436	422	46.9	6.8
	Total	30,384	4883	2134	2116	43.7	7.0
	CI					0.0312	− 0.0146
2011	I	5678	911	436	490	47.9	8.6
	II	5839	813	397	396	48.8	6.8
	III	5815	886	446	410	50.3	7.1
	IV	5682	726	404	394	55.6	6.9
	V	5872	562	315	365	56.0	6.2
	Total	28,886	3898	1998	2055	51.3	7.1
	CI					0.0248	− 0.0540
2012	I	5919	925	400	538	43.2	9.1
	II	6127	893	387	405	43.3	6.6
	III	5932	788	360	379	45.7	6.4
	IV	6125	818	388	410	47.4	6.7
	V	6480	816	373	431	45.7	6.7
	Total	30,583	4240	1908	2163	45.0	7.1
	CI					0.0139	− 0.0566
2015	I	5083	710	297	584	41.8	11.5
	II	6280	683	322	565	47.1	9.0
	III	5357	605	294	454	48.6	8.5
	IV	5665	551	251	424	45.6	7.5
	V	6312	750	355	524	47.3	8.3
	Total	28,697	3299	1519	2551	46.0	8.9
	CI					0.0151	− 0.0791
2019	I	4771	804	405	624	50.4	13.1
	II	4780	597	292	501	48.9	10.5
	III	3791	501	240	412	47.9	10.9
	IV	5676	888	423	698	47.6	12.3
	V	4803	665	316	539	47.5	11.2
	Total	23,821	3455	1676	2774	48.5	11.6
	CI					− 0.0105	− 0.0073

**Figure 3:**
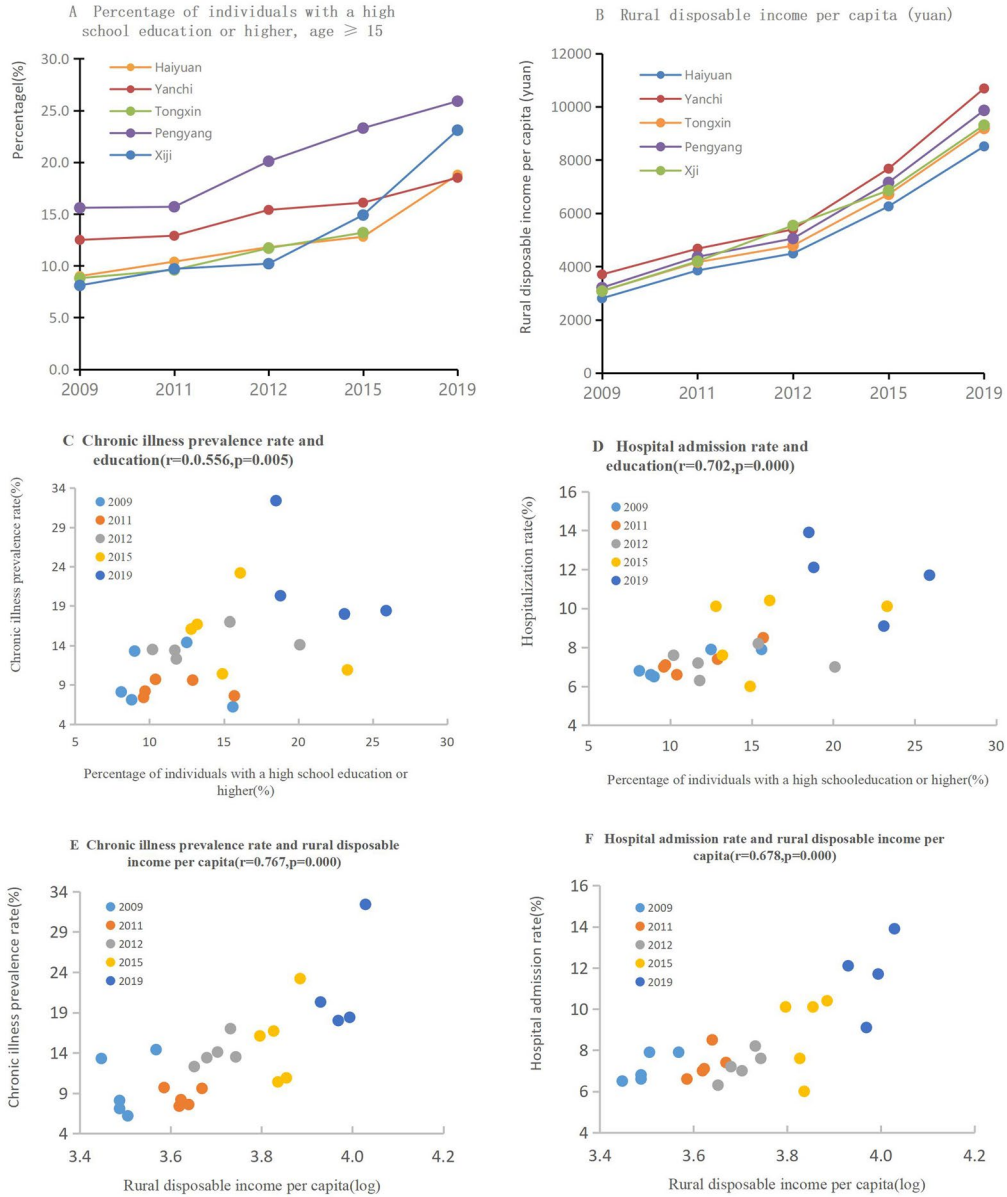
The associations between the chronic illness prevalence rate or the hospitalization rate and education levels or rural disposable income per capita.

Figure 3 and Table 2 show the associations between the chronic illness prevalence rate or the hospitalization rate and socioeconomic status (rural education levels or rural per capita disposable income). Educational levels improved from 2009 to 2019 among people aged over 15 years old, with a substantial increase in the number of people with a mid-level education in each county. The percentage of individuals who had received an education above the high-school level among those over 15 years old in Pengyang County was the highest, and Xiji County has experienced the fastest increase in the proportion of individuals with a high school education or above over the past 10 years. The chronic illness prevalence rate and the hospitalization rate were positively associated with rural per capita disposable income and the proportion of people with at least a senior high school education (P < 0.01, Fig. 3). The multivariate regression (Table 2) shows that rural disposable income per capita is a protective factor for the rate of receiving a medical consultation in the last 2 weeks and the rate of hospital admission (both P < 0.0001). The percentage of individuals with a high school education or higher was negatively associated with the rate of receiving a medical consultation in the last 2 weeks (P < 0.05). The chronic illness prevalence rate was positively associated with the rate of receiving a medical consultation in the last 2 weeks (P < 0.05).

**Table 2:** Associations between rural disposable income per capita, education levels, the chronic illness prevalence rate and the medical consultation rate/hospitalization rate.

	The medical consultation rate			The hospitalization rate		
	β (95% CI)	SE	P	β (95% CI)	SE	P
Rural disposable income per capita	0.008 (0.005, 0.011)	0.0002	0.000	0.001 (0.0004, 0.0014)	0.0002	0.000
High school education or higher (%)	− 2.58 (− 4.252, 0.884)	0.859	0.003	− 0.197 (− 0.441, 0.046)	0.124	0.113
Chronic illness prevalence rate	1.667 (0.903, 2.431)	0.389	0.000	0.024 (− 0.092, 0.141)	0.596	0.681

## Discussion

In the 10 years since the launch of the healthcare reform, China has made steady progress towards achieving its reform goals^11^. The utilization of health services has increased year by year. After the reform, more investments were made, and the accessibility of health services was increased, so health services were improved. The use of services has increased, which is consistent with the results of others^17–21^. In 2012, the number of insured persons reached 1.3 billion^22–24^. Access to health insurance is conductive to the use of health services^25^.

The hospitalization rate among rural residents in the project counties was higher than that in the control counties, which may be due to the adjustments in the medical insurance policy in the project counties. The chronic disease prevalence rate among rural residents of Ningxia was lower than that reported in the Sixth National Health Service Survey (35.2%)^17^. There may be two reasons for the rapid rise in the incidence rate of chronic diseases: first, the improvement of accessibility of health services has increased the number of residents seeking medical services; second, the policy of free physical examination for elderly has resulted in more chronic diseases being discovered. Chronic diseases have become the most important diseases affecting residents' health^26,27^. We must improve the prevention and treatment of chronic diseases. The health needs of older people are much higher than those of young people, which should be fully considered by decision makers when formulating health service reform policies.

From 2009 to 2019, equity in the utilization of health services has improved. The utilization of health services has become increasingly equitable. These results are consistent with existing empirical studies on access and equal access to healthcare^28–32^. This result shows that medical and healthcare reform has played a positive role in increasing the utilization of health services among rural residents, especially within the low-income groups. Compared with the improvement in the fairness of outpatient health service utilization, the fairness in inpatient service utilization needs attention. In this case, given the high cost of hospitalization, some residents choose to replace hospitalization with outpatient services even if the disease is serious. The hospitalization costs at the high-quality hospitals that can provide high-quality inpatient medical services are relatively high, which limits the utilization of inpatient medical services by low-income residents to a certain extent^33^.

The higher one’s education level is, the lower that person’s utilization of outpatient health services is. One possible explanation is that most of the people with higher education levels are young people with better health and greater health awareness, and the prevalence of illness within this group is lower. The other explanation is that people with higher education levels choose to self-treat minor diseases. The trends in the chronic disease prevalence rate and the rate of visiting a medical professional in the last 2 weeks and the directions of those trends were consistent; the higher the prevalence of chronic diseases was, the higher the rate of outpatient health service utilization. This is in line with studies that have reported that having a chronic condition is a strong predictor of healthcare utilization^34,35^. This finding is also in line with studies performed in Ghana, which have reported that having multiple chronic conditions is a predictor of health service utilization^36^.

## Conclusion

This study showed that China’s 2009 healthcare reform had a positive influence on the rate of and equity in health service utilization. We should pay attention to strengthen the prevention and treatment of chronic diseases, focus attention on the health status of elderly residents and improve social-economic conditions, especially the level of education.

## Data Availability

The data that support the findings of this study are available from Ningxia Medical University, but restrictions apply to the availability of these data, which were used under licence for the current study and so are not publicly available. Data are, however, available from the submitting authors upon reasonable request and with permission of Ningxia Medical University.
